# The Psychosocial Influence of Companion Animals on Positive and Negative Affect during the COVID-19 Pandemic

**DOI:** 10.3390/ani11072084

**Published:** 2021-07-13

**Authors:** Lori R. Kogan, Jennifer Currin-McCulloch, Cori Bussolari, Wendy Packman, Phyllis Erdman

**Affiliations:** 1 Clinical Sciences, Colorado State University, Fort Collins, CO 80523, USA; 2 School of Social Work, Colorado State University, Fort Collins, CO 80523, USA; jen.currin-mcculloch@colostate.edu; 3 Counseling Psychology, University of San Francisco, San Francisco, CA 94117, USA; bussolari@usfca.edu; 4 Department of Psychology, Palo Alto University, Palo Alto, CA 94304, USA; wpackman@paloaltou.edu; 5 College of Education, Washington State University, Pullman, WA 99163, USA; perdman@wsu.edu

**Keywords:** COVID-19, companion animal, dog, cat, psychosocial, depression, anxiety

## Abstract

**Simple Summary:**

The initial months of COVID-19 forced people to quickly adapt to dramatic changes to their daily lives. As a result of the inevitable decrease in access to social support available during the lockdown phase of COVID-19, countless individuals relied upon their companion dogs and cats. Given the strong connections many people have with their companion animals, this study hypothesized that companion dogs and cats would positively impact guardians’ mental health. Anonymous online surveys were used to test this premise. A total of 5061 responses, primarily females (89%) from the United States (84%), were analyzed. Results suggest that companion animals played a critical role in reducing feelings of depression, anxiety, isolation, and loneliness. Companion animals also helped increase guardians’ experiences of self-compassion, ability to maintain a regular schedule, feel a sense of purpose and meaning, and cope with uncertainty. This was most prevalent for women under the age of 40 who were highly bonded to their companion animal. In conclusion, our study suggests that a companion dog or cat can help mitigate the effects of extreme stress and social isolation.

**Abstract:**

The initial months of COVID-19 forced people to quickly adapt to dramatic changes to their daily lives. As a result of the inevitable decrease in access to social support available during the lockdown phase of COVID-19, countless individuals relied upon their companion dogs and cats. Given the strong connections people often have with their companion animals, this study hypothesized that companion dogs and cats would positively impact guardians’ mental health. Anonymous, cross-sectional online surveys were used to test this premise. A total of 5061 responses, primarily females (89%) from the United States (84%), were analyzed. Results suggest that companion animals played a critical role in helping reduce feelings of depression, anxiety, isolation, and loneliness for a majority of pet guardians. Companion animals also helped increase guardians’ experiences of self-compassion, ability to maintain a regular schedule, feel a sense of purpose and meaning, and cope with uncertainty. This was most pronounced for women under the age of 40 who were highly bonded to their companion animal. In conclusion, our study suggests that a companion dog or cat can buffer the effects of extreme stress and social isolation as witnessed during the COVID-19 pandemic.

## 1. Introduction

The coronavirus disease 2019 (COVID-19) was initially reported in December 2019 in Wuhan, China, and within months manifested into a global pandemic [1]. Fears intensified as the number of cases grew exponentially and scientific knowledge of the means of transmission was limited and contradictory [2]. To mitigate the potential spread of COVID-19, numerous governmental agencies issued public health mandates requiring social distancing and the wearing of masks [3]. These first months of COVID-19 created numerous stressors for many, including social isolation, fear of exposure, and job insecurity, creating a collective emotional contagion of pervasive anxiety [4,5].

Efforts to contain the virus forced people to quickly adapt to dramatic changes to their daily lives. Social interaction restrictions required many businesses—including healthcare services and veterinary offices—to quickly shift their mode of operations to remote settings or alternative services. Quarantine guidelines resulted in social isolation from family, friends, coworkers, and many of the activities that nurture coping capacities. Consequently, pandemic-related interruptions to daily lives, perceived loss of personal control, collective grief, and quarantine mentality resulted in a surge of mental health crises [6,7,8]. Communities across the globe experienced soaring rates of anxiety, depression, loneliness, post-traumatic stress, and suicidal ideations [9,10,11]. For example, a survey in November 2020 that included over 1.4 million US adults, discovered anxiety and depression at rates six times higher compared to norms for early 2019 [12]. Elevated rates of anxiety, depression, and loneliness during this time were reported in regions worldwide [7,13,14,15,16,17,18]. To address the surge in mental health distress, new forms of mental health services were created, including digital mental health tools (mobile applications, websites, etc.) and phone-based or text-based crisis lines [12,19] Yet, even with these additional options, the high demand for mental health services resulted in large numbers of individuals reporting unmet needs [12,20].

The deleterious impact of COVID-19 related concerns varied among individuals, based partially on social determinants of health and mental health such as age, gender, race/ethnicity, income, education, and previous mental and physical health crises. Commonly cited predictors of COVID-19 mental distress are female gender, younger age (18–39), lower socioeconomic status, limited access to social support, and high fear levels of COVID-19 contagion [21,22,23]. Pèrez et al. [24], for example, found the most prevalent predictors of COVID-19 related distress included gender, age, and living alone. Other studies also found that young adults aged 18–39, compared to older adult populations, experienced elevated levels of COVID-19 related anxiety and depression [14,16,25,26]. It has been suggested this might be because young adults have less previous exposure to trauma and have therefore not developed the coping skills needed to adapt to pandemic-related stressors [14]. Additionally, Glowacz and Schmits [25] posited that young adults may be more at risk for mental distress due to smaller living spaces and higher intolerance of uncertainty. Others proclaim that young adults’ extended time on social media may increase their exposure to distressing news stories [16]. Sewall et al. [27], however, suggested that pandemic-related stresses such as job loss and roommate conflicts may be more likely to create emotional distress and suicidal ideation than increased screen time and exposure to social media. Regardless of the reasons behind the increased distress for younger individuals, this negative impact appears most pronounced for young females [12,15,28]. 

In addition to age, loneliness and a perceived lack of social support during the initial months of COVID-19 have been associated with increased depression [26,29]. However, it would appear that older adults, and people with a stronger sense of purpose, were better able to cope with the imposed limits to social interactions [30]. With the lack of access to social support networks during the lockdown phase of COVID-19, many individuals relied on their companion dogs and cats to fill this void [31]. Prior to the pandemic, there were over 90 million companion dogs [32] and 58 million companion cats [33] in the United States. Attachment theory suggests that the bonds that individuals create with companion animals are important for security, safety, and decreased loneliness [34,35,36], and a growing body of research supports the premise that companion animals offer numerous benefits and that humans bond with nonhuman animals in ways that are comparable with human–human attachment [37,38,39,40,41,42,43]. It is not surprising, therefore, that companion animals are often seen as part of the family or even as surrogate children [38,44]. In fact, people consider their relationships with their dog or cat profoundly important and they grieve deeply when their companion animal dies [45,46,47].

Research conducted during the initial months of COVID-19 has shown that while many companion animal guardians were worried about being able to provide for their companion animal and access veterinary medical services [48,49,50,51,52,53,54], they were grateful for the increased quality time with them and reflected on how their companion animal offered a decreased sense of loneliness [49,54,55]. Given the strong connections people have with their companion animals, and the potential benefits of companion dogs and cats, this study hypothesized that companion dogs and cats would positively impact guardians’ mental health. Therefore, the main aims of the study were (1) to discover the impact of companion cats and dogs on their guardians’ COVID-19 related feelings; and (2) to determine how sociodemographic, psychosocial, or human–animal attachment factors may help predict this impact on psychosocial adaptation to COVID-19.

## 2. Materials and Methods

Two online, anonymous, cross-sectional surveys were developed using Qualtrics (Qualtrics, Inc., Provo, UT, USA): one for dog guardians and one for cat guardians (see Appendix A for the cat guardian survey). The surveys were designed and reviewed by the co-investigators and tested by cat and dog guardians. Testing included assessment for ambiguity and/or potentially missing or inappropriate response options. Revisions were made based on the results of the pilot testing, and the final surveys (see Appendix A for the dog survey) were approved by the lead University’s Institutional Review Board (IRB # 20-10003H). Survey respondents were recruited through social media outlets and human–animal focused organizations from March to May, 2020. 

This paper focuses on the survey questions pertaining to the impact of dogs and cats on guardians’ COVID-related feelings. Other aspects of the study (e.g., changes in time spent with companion animals and the impact of these changes; concerns related to caring for a companion animal, veterinary care, and the ability to obtain and afford pet supplies) have been reported previously [50,51]. Participants included an international sample of cat and dog guardians over the age of 18. For people who indicated they had more than one cat or dog, they were instructed to answer the survey questions for their companion animal whose name starts with a letter closest to the beginning of the alphabet (“for example, choose to answer about Fluffy rather than Sylvester since ’F‘ comes before ’S‘ in the alphabet”). Demographic data were collected (e.g., age group, gender, country of residence, current level of COVID restrictions, current level of social support, and number of adults and children living in the home). Respondents were asked to rate their bond with their cat or dog on a scale from 1 (not at all bonded) to 10 (extremely bonded). For analysis purposes, bond scores were categorized into low (1–5), medium (6–8) and high (9–10). Participants were then asked to report if/how their cat or dog had impacted feelings they might have related to COVID-19 (“Please indicate how your cat/dog has impacted each of the following feelings you might be having related to COVID-19”) on a five-point Likert scale from 1 = greatly decreased to 5 = greatly increased. They could also select “Not Applicable” if that feeling was not an issue for them. For analyses, these results were used to create three levels: decreased the feeling, had no impact, or increased the feeling. For reporting purposes, these feelings were then categorized into negative feelings (i.e., anxiety, depression, overwhelmed, isolated, and lonely) and positive feelings (i.e., ability to maintain a regular schedule, ability to cope with uncertainty, give purpose/meaning to one’s life, and compassion towards oneself). 

Data were analyzed using SPSS (IBM, Armonk, NY, USA). Descriptive statistics were calculated to characterize the impact of dogs and cats on COVID-19 related feelings. Because all participants did not answer every question, the number of responses for each item assessed are noted. Initial analysis revealed no species difference on companion animals’ impact of guardians’ COVID-19 related feelings; therefore, dog and cat guardian responses were combined for overall companion animal impact analyses. Binary regression was performed on each of the nine assessed feelings related to COVID-19 after conducting Pearson correlations to determine the potential predictive factors to include in each regression equation. Possible predictive factors included animal species (dog or cat), guardian age (39 and younger, 40–59 years, 60 years of age and older), guardian gender (male or female), bond score (low, medium, high), and living situation (living alone or living with other adults). Due to multiple regression analyses, Bonferroni correction was used, with the resulting *p* value accepted as statistically significant at 0.006.

## 3. Results

### 3.1. Descriptive Statistics 

The total number of responses was 5061, with most participants being female (4485; 89.0%) and living with at least one other adult (3850, 76.1%) and having no children under the age of 18 living in the home (4173, 82.5%). Most participants were from the United States (4228, 83.6%) and at the time of completing the survey, most reported restrictions in their city at the level of ”all non-essential stores/businesses closed and ordered/strongly recommended to stay at home” (3988, 78.8%). Ages included those 39 years and younger (1508, 30.2%), 40–59 years of age (2153, 43.1%), and those 60 years of age and older (1335, 26.7%) (Table 1).

### 3.2. Guardian/Animal Bond and Social Support

Guardians were asked to report their bond level with their companion animal both one month before COVID-19 and ”currently” on a scale of 1 (not bonded at all) to 10 (extremely bonded). The mean score for one month before COVID-19 was 9.02 (SD 1.42), significantly lower than “currently” (9.34, SD 1.14; t = 24.55 *p* < 0.001). Perceived level of social support, both immediately before COVID-19 and “currently” was also assessed, with many participants reporting a reduction in social support (X^2^ = 1527.24(4), *p* < 0.001) (Table 2). 

### 3.3. COVID-19 Related Feelings

**Figure 1:**
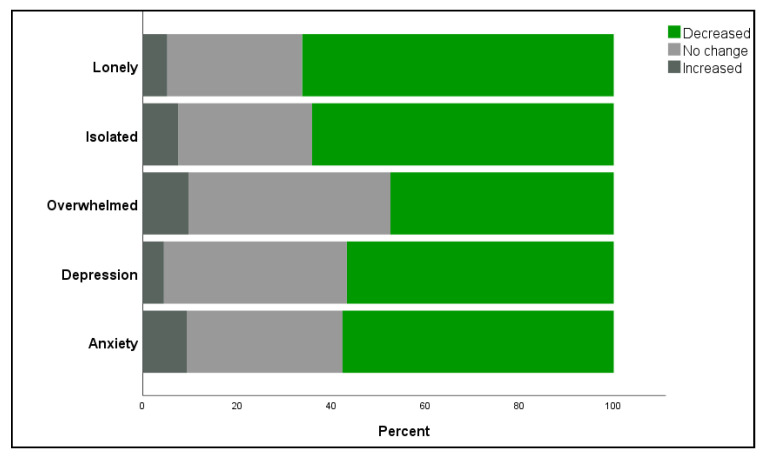
Reported impact of companion animals on COVID-19 related negative feelings.

**Figure 2:**
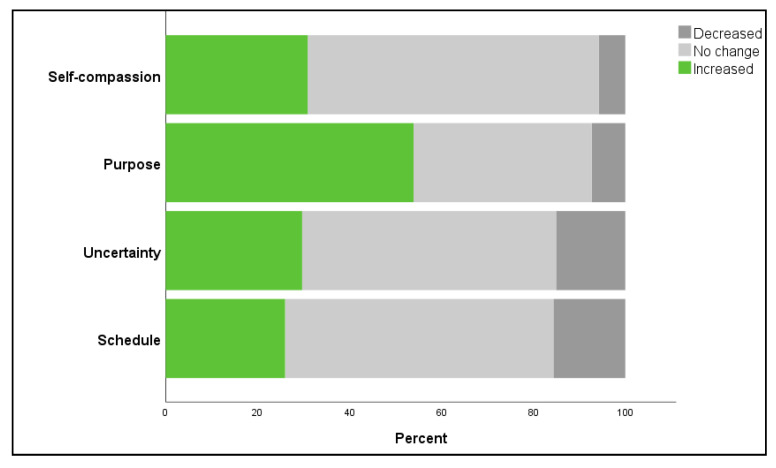
Reported impact of companion animals on COVID-19 related positive feelings.

Guardians were asked to report the impact they felt their companion animals had on their COVID-19 related feelings. When assessing negative feelings, the majority of guardians reported that their companion animals helped decrease feelings of anxiety (2655, 57.6%), depression (2476, 56.7%), isolation (2945, 64%), overwhelmed (2151, 47.4%), and loneliness (3029, 66.2%). For positive feelings, 2540 (53.9%) of participants reported their companion animals helped give them purpose or meaning to their life and 26% reported they helped them maintain a regular schedule. Approximately 30% reported their companion animals helped with their ability to cope with uncertainty and feelings of self-compassion (Table 3, Figure 1 and Figure 2).

Prior to running binary regression on the negative and positive emotions/behaviors related to COVID-19, Pearson correlations were calculated for each of the nine emotions and potential predictive factors (see Table 4 for negative feelings and Table 5 for positive feelings). For each emotion, all correlated factors were entered into a binary regression model to determine the best predictors for each independent variable.

### 3.4. Binary Regression Models—Negative Emotions

#### 3.4.1. Anxiety

The binary regression model predicting anxiety was significant (X^2^ = 167.93(5), *p* < 0.001), with an *R*^2^ of 0.045. Significant predictors of the reported impact of a companion animal on anxiety included guardian gender (*p* < 0.001), guardian age (*p* < 0.001), and bond level (*p* < 0.001). Female guardians were 1.5 times more likely to report that their companion animal helped decrease their anxiety than male guardians. Guardians 40 years of age and older were approximately half as likely to report a positive impact as guardians 39 years old or younger. Additionally, the odds of a companion animal having a positive impact on anxiety were 1.5 times higher for those who reported a medium level bond and almost 2 times higher for those with a strong bond compared to those who reported a low bond. Bond level was the strongest predictor of a reduction in anxiety (Table 6).

#### 3.4.2. Depression

When assessing the independent variable, feelings of depression, the binary regression model was significant (X^2^ = 186.86(7), *p* < 0.001), with an *R*^2^ of 0.049. Significant predictors of the reported impact of a companion animal on depression included guardian gender (*p* < 0.001), guardian age (*p* < 0.001), and bond level (*p* < 0.001). Female guardians were more likely to report that their companion animal helped decrease their depression than male guardians. Guardians 39 years old or younger were nearly twice as likely to report a positive impact of companion animals compared to guardians 40 years of age and older. The odds of their companion animal having a positive impact on depression were about 2 times higher for those with a strong bond with their companion animal when compared to those who reported a low bond. Additionally, guardians living with other adults were less likely to report their companion animals helped with depression than those living alone. Species was not a significant predictor. Bond level was the strongest predictor of reduced depression (Table 6).

#### 3.4.3. Overwhelmed

The binary regression model predicting feeling overwhelmed was significant (X^2^ = 133.05(6), *p* < 0.001), with an *R*^2^ of 0.036. Significant predictors of a companion animal on feeling overwhelmed included guardian gender (*p* < 0.001), guardian age (*p* < 0.001), and bond level (*p* < 0.001). Female guardians were approximately 1.5 times more likely to report that their companion animal helped decrease their feelings of being overwhelmed than male guardians, while guardians 40 years of age and older were approximately half as likely to report a positive impact as younger guardians. Additionally, bond level was the strongest predictor; those with a strong bond were more than 3 times as likely to report their companion animals had a positive impact on feeling overwhelmed compared to those with a low bond. Species was not a significant predictor (Table 6).

#### 3.4.4. Isolation

When assessing feelings of isolation, the binary regression model was significant (X^2^ = 194.85(7), *p* < 0.001), with an *R*^2^ of 0.052. Significant predictors of the reported impact of a companion animal on feeling isolated included guardian gender (*p* < 0.001), guardian age (*p* < 0.001), bond level (*p* < 0.001), species (*p* < 0.001), and living situation (*p* < 0.001). Female guardians were more likely to report that their companion animal helped decrease their feelings of isolation than male guardians. Guardians 40 years of age and older were approximately 40–50% less likely to report a positive impact than guardians 39 years old or younger. Additionally, the odds of a companion animal having a positive impact on feelings of isolation were 1.3 times higher for those who reported a medium level bond and over 2 times higher for those with a strong bond compared to those who reported a low bond. Those living with others were less likely to report a positive impact on isolation compared to those living alone. Lastly, those with a dog were more likely to report positive impact than those with a cat (Table 6).

#### 3.4.5. Loneliness

For the independent variable loneliness, the binary regression model was significant (X^2^ = 219.92(6), *p* < 0.001), with an *R*^2^ of 0.059. Significant predictors of the reported impact of a companion animal on loneliness included guardian gender (*p* < 0.001), guardian age (*p* < 0.001), and bond level (*p* < 0.001). Female guardians and those 39 years old or younger were more likely to report that their companion animal helped decrease their loneliness than male guardians or those 40 years of age and older. Additionally, those with a high bond were over 3 times as likely to report their companion animals helped with loneliness when compared to those who reported a low bond. Species was not a significant predictor (Table 6).

### 3.5. Binary Regression Models—Positive Emotions

#### 3.5.1. Maintaining a Schedule

The binary regression model predicting the ability to maintain a regular schedule was significant (X^2^ = 135.97(4), *p* < 0.001), with an *R*^2^ of 0.041. Significant predictors of the reported impact of a companion animal on maintaining a regular schedule included guardian gender (*p* < 0.001), guardian age (*p* < 0.001), and species (*p* < 0.001). Female guardians were about 1.7 times more likely to report that their companion animal helped them maintain a regular schedule than male guardians. Guardians 40 years of age and older were approximately 50–75% less likely to report a positive impact than guardians 39 years old or younger. Lastly, those with a dog were twice as likely to report positive impact on the ability to maintain a regular schedule as those with a cat (Table 7).

#### 3.5.2. Dealing with Uncertainty

When assessing companion animals’ impact on guardians’ ability to deal with uncertainty, the binary regression model was significant (X^2^ = 52.59(6), *p* < 0.001), with an *R*^2^ of 0.015. Significant predictors of a companion animal’s impact included guardian gender (*p* < 0.001), guardian age (*p* < 0.001), and bond level (*p* < 0.001). Female guardians and those guardians 39 years and younger were more likely to report their companion animals helped them to deal with uncertainty than male guardians or those 40 years old or older. Bond level and species were not significant predictors (Table 7).

#### 3.5.3. Give a Sense of Purpose or Meaning to Life

The binary regression model predicting companion animals’ ability to give guardians a sense of purpose or meaning to life was significant (X^2^ = 105.30(6), *p* < 0.001), with an *R*^2^ of 0.028. Significant predictors of the reported impact of a companion animal included guardian gender (*p* < 0.001), guardian age (*p* < 0.001), bond level (*p* < 0.001), and living situation (*p* < 0.001). Female guardians and those younger than 40 were more likely to report that their companion animal helped give them purpose than male guardians or those 40 and older. Additionally, the odds of companion animals having a positive impact on life purpose were 1.4 times higher for those who reported a medium level bond and almost 3 times higher for those with a strong bond compared to those who reported a low bond. Those living with other adults were less likely to report a positive impact on life purpose compared to those living alone (Table 7).

#### 3.5.4. Self-Compassion

For the independent variable, self-compassion, the binary regression model was significant (X^2^ = 56.36(4), *p* < 0.001), with an *R*^2^ of 0.016. Significant predictors of the reported impact of a companion animal on self-compassion included guardian gender (*p* < 0.001), guardian age (*p* < 0.001), and species (*p* < 0.001). Female guardians and those 38 years old and younger were more likely to report that their companion animal helped increase self-compassion compared to male guardians or those aged 40 and older. Species was not a statistically significant predictor (Table 7).

## 4. Discussion

This study was conducted during the initial months of the COVID-19 pandemic. During this phase, many were specifically instructed to stay at home as much as possible and limit their in-person interactions. The externally imposed social isolation, along with the fear of acquiring COVID-19 and uncertainty about the future, resulted in increased psychological stress, loneliness, and anxiety for many people [1,18,56,57,58]. 

Prior to COVID-19, research highlighted how animals may reduce stress [59], help support people with chronic mental health difficulties [60], increase positive emotions [61], and reduce loneliness [62,63]. In the current study, we found that for many companion animal guardians, companion animals played a critical role in helping reduce feelings of depression, anxiety, isolation, and loneliness. They also helped increase feelings of self-compassion, their ability to maintain a regular schedule, feel a sense of purpose and meaning, and cope with uncertainty. Perhaps it is not surprising therefore, that many people reported feeling more bonded with their companion animal during the initial months of COVID-19 than before the virus. Other studies conducted during the COVID-19 lockdown phase also suggest that companion dogs and cats had a positive impact on guardians’ mental and physical functioning, highlighting the role of companion animals as social buffers for psychological distress and loneliness [52,53]. 

Yet, the benefits of companion animals during the initial months of COVID-19 do not appear equal for all companion animal guardians. Our findings extend and refine our understanding of these positive outcomes. Of significant interest, women under the age of 40 who were highly bonded to their companion animal appear to reap the greatest mental health benefits. Reasons for these results are not known, but perhaps women, who have been shown to display more empathy and positive attitudes towards companion animals than men [64], are more likely to prioritize their relationship with their companion animal. Conceivably, younger women, who appear to have struggled more with COVID-19 related stressors than others [15], were more likely to look to their companion animals for support during such a highly stressful time. While it is possible that the stressors of aging, and the fear of getting ill and not being able to care for one’s companion animal, impacted the extent to which older participants found their companion animals helpful, we suggest an alternative reason. Perhaps the increased resilience found to be present among older adults while isolated during the pandemic [65] led to a decreased need for psychological support from their companion animals. During early COVID-19 months, older age was associated with better mental health scores [16,22]. In other words, older adults may not have needed as much support from their cat or dog as younger guardians, who perhaps had yet to develop the levels of resilience and coping strategies that come with time and life experience.

### Future Directions and Limitations

With respect to study limitations, while significant, the low R^2^ of the regression models likely reflects the high variability among cat and dog guardians and suggest that other intrinsic and extrinsic factors contributed to the impact that companion animals played in their guardians’ COVID-19 related feelings. Additional research to help determine these nuances are needed. Other study limitations include the cross-sectional nature of the design and the self-selection process for participants, resulting in primarily White women from the United States. The experiences of cat and dog guardians from other countries and at different periods during the pandemic may differ based on their community’s COVID exposure and public health precautions. Therefore, generalization of the current study is limited by the underrepresentation of non-female self-identified US participants and an overrepresentation of those who may have highly attached relationships with their companion dog or cat. 

## 5. Conclusions

In conclusion, our study supports the premise that during an unparalleled time of crisis, stress, and social isolation, a companion dog or cat can offer a plethora of mental health benefits. This is especially the case for younger women with a strong bond to their companion animal. 

## Figures and Tables

**Table 1:** Participant sociodemographics (*n* = 5061).

Category	*n* (%)
Species (*n* = 5061)	
Dog guardians	4105 (81%)
Cat guardians	956 (19%)
Gender (*n* = 5039)	
Female	4485 (89%)
Male	517 (10%)
Non-binary	37 (1%)
Country (*n* = 5059)	
United States	4228 (84%)
Canada	359 (7%)
United Kingdom	185 (4%)
Australia	66 (1%)
Other	221 (4%)
Age (*n* = 4996)	
Under 39	1508 (30%)
40–59	2153 (43%)
60 and older	1335 (27%)
Living arrangement (*n* = 5061)	
Live with no other adult in home	1211 (24%)
Live with at least one other adult in home	3850 (76%)
Children living in the home (*n* = 5061)	
None	4173 (83%)
One	488 (10%)
More than one	400 (8%)
Dogs and/or cats living in the home	
One	2322 (46%)
Two	1604 (32%)
Three	613 (12%)
Other	522 (23%)
Restriction level	
Nonessential businesses closed; ordered to stay at home	3988 (79%)
Nonessential businesses closed; not ordered to stay at home	855 (17%)
Some stores/businesses and restaurants closed	174 (3%)
No current restrictions	24 (1%)
Other	20 (<1%)

**Table 2:** Perceived social support immediately prior to COVID-19 and at the time of the survey.

Category	Pre-COVID-19	Post-COVID-19
Minimal available social support	431 (9%)	1459 (29%)
Some available social support	1973 (39%)	2248 (44%)
Extensive available social support	2657 (53%)	1354 (27%)

**Table 3:** Companion animal impact on COVID-19 related feelings.

Category	Decreased	No Change	Increased
Anxiety (*n* = 4613)	2655 (58%)	1526 (33%)	432 (9%)
Depression (*n* = 4370)	2476 (57%)	1697 (39%)	197 (5%)
Feeling overwhelmed (*n* = 4538)	2151 (47%)	1944 (43%)	443 (10%)
Feeling isolated (*n* = 4600)	2945 (64%)	(1307 (28%)	348 (8%)
Feeling lonely (*n* = 4578)	3029 (66%)	1310 (29%)	239 (5%)
Maintain regular schedule (*n* = 4632)	725 (16%)	2704 (58%)	1203 (26%)
Ability to cope with uncertainty (*n* = 4598)	693 (15%)	2540 (55%)	1365 (30%)
Give purpose/meaning (*n* = 4710)	7%	39%	54%
Compassion towards myself (*n* = 4635)	6%	63%	31%

**Table 4:** Correlation matrix of negative emotions and potential predictive variables: social support, species, age, gender, bond, and living arrangement.

	Anxiety	Depression	Overwhelmed	Isolated	Lonely	Social Support	Species	Age	Gender	Bond	Living Arrangement
Anxiety	1										
Depression	0.634 **	1									
Overwhelmed	0.597 **	0.575 **	1								
Isolated	0.533 **	0.544 **	0.508 **	1							
Lonely	0.508 **	0.542 **	0.464 **	0.800 **	1						
Social support	0.004	−0.018	0.041 **	−0.002	−0.009	1					
Species	−0.027	−0.030 *	−0.031 *	−0.064 **	−0.039 **	0.007	1				
Age	−0.129 **	−0.144 **	−0.109 **	−0.163 **	−0.182 **	−0.017	0.148 **	1			
Gender	0.067 **	0.047 **	0.047 **	0.043 **	0.068 **	0.034 *	0.040 **	−0.002	1		
Bond	0.089 **	0.084 **	0.082 **	0.038 **	0.048 **	0.055 **	0.098 **	0.119 **	0.009	1	
Living arrangement	−0.004	−0.033 *	0.009	−0.034 *	−0.023	0.080 **	−0.130 **	−0.140 **	−0.027	−0.049 **	1

** = *p* < 0.01 level (2-tailed); * = *p* < 0.05 level (2-tailed).

**Table 5:** Correlation matrix of positive emotions and potential predictive variables: social support, species, age, gender, bond, and living arrangement.

	Schedule	Uncertainty	Purpose	Self-Compassion	Species	Social Support	Bond	Gender	Age	Living Arrangement
Schedule	1									
Uncertainty	0.298 **	1								
Purpose	0.224 **	0.338 **	1							
Self-compassion	0.184 **	0.314 **	0.398 **	1						
Species	−0.090 **	−0.028 *	−0.009	0.004	1					
Social support	0.016	0.007	−0.022	0.027	0.007	1				
Bond	−0.023	0.040 **	0.065 **	0.053 **	0.098 **	0.055 **	1			
Gender	0.056 **	0.046 **	0.052 **	0.037 **	0.040 **	0.034 *	0.009	1		
Age	−0.135 **	−0.075 **	−0.097 **	−0.098 **	0.148 **	−0.017	0.119 **	−0.002	1	
Living arrangement	−0.012	−0.018	−0.042 **	−0.023	−0.130 **	0.080 **	−0.049 **	−0.027	−0.140 **	1

** = *p* < 0.01 level (2-tailed); * = *p* < 0.05 level (2-tailed).

**Table 6:** Binary regression for negative COVID-19 related feelings.

Independent Variable	Potential Predictive Factors	OR	95% CI	*p*-Value
Anxiety				
	Gender			
	Female	1.57	(1.30, 1.89)	<0.001
	Male *			
	Age			
	≤39 *			
	40–59	0.595	0.571, 0.683	<0.001
	≥60	0.462	0.397, 0.539	<0.001
	Bond			
	Low *			
	Medium	1.688	1.441, 1.976	<0.001
	High	3.810	1.902, 7.632	<0.001
Depression				
	Gender			
	Female	1.37	(1.13, 1.65)	=0.001
	Male *			
	Age			
	≤39 *			
	40–59	0.550	(0.477, 0.634)	<0.001
	≥60	0.423	(0.362, 0.495)	<0.001
	Bond			
	Low *			
	Medium	1.64	1.40, 1.92	<0.001
	High	4.29	2.04, 9.03	<0.001
	Living arrangement			
	Live with others	0.78	0.68, 0.89	<0.001
	Live alone *			
	Species			
	Cat	0.87	0.75, 1.01	=0.064
	Dog *			
Overwhelmed				
	Gender			
	Female	1.38	1.14, 1.67	<0.001
	Male *			
	Age			
	≤39 *	0.59	0.51, 0.68	<0.001
	40–59	0.53	0.45, 0.61	<0.001
	≥60			
	Bond			
	Low *			
	Medium	1.69	0.144, 1.99	<0.001
	High	3.25	1.55, 6.82	<0.001
	Species			
	Cat	0.86	0.74, 1.00	=0.043
	Dog *			
Isolation				
	Gender			
	Female	1.34	1.11, 1.62	=0.002
	Male *			
	Age			
	≤39 *			
	40–59	0.54	0.47, 0.63	<0.001
	≥60	0.39	0.33, 0.46	<0.001
	Bond			
	Low *			
	Medium	1.31	1.12, 1.54	= 0.001
	High	2.28	1.23, 4.24	= 0.009
	Living arrangement			
	Live with others	0.73	0.63, 0.84	<0.001
	Live alone *			
	Species			
	Cat	0.74	0.64, 0.86	<0.001
	Dog *			
Lonely				
	Gender			
	Female	1.58	1.31, 1.91	<0.001
	Male *			
	Age			
	≤39 *			
	40–59	0.54	0.47, 0.63	<0.001
	≥60	0.35	0.30, 0.42	<0.001
	Bond			
	Low *			
	Medium	1.40	1.19, 1.64	<0.001
	High	3.13	1.66, 5.90	<0.001
	Species			
	Cat	0.89	0.77, 1.03	=0.119
	Dog *			

* referent; OR, odds ratio; CI, confidence interval; bold denotes statistical significance.

**Table 7:** Binary regression for negative COVID-19 related feelings.

Independent Variable	Potential Predictive Factors	OR	95% CI	*p*-Value
Schedule				
	Gender			
	Female	**1.69**	**1.32, 2.16**	**<0.001**
	Male *			
	Age			
	≤39 *			
	40–59	**0.75**	**0.63, 0.89**	**<0.001**
	≥60	**0.47**	**0.39, 0.57**	**<0.001**
	Species			
	Cat	**0.59**	**0.48, 0.71**	**<0.001**
	Dog *			
Uncertainty				
	Gender			
	Female	**1.43**	**1.15, 1.79**	**<0.001**
	Male *			
	Age			
	≤39 *			
	40–59	**0.76**	**0.65, 0.90**	**0.001**
	≥60	**0.64**	**0.54, 0.76**	**<0.001**
	Bond			
	Low *			
	Medium	1.26	1.05, 1.51	0.019
	High	3.08	1.21, 7.86	0.012
	Species			
	Cat	0.85	0.72, 1.01	=0.065
	Dog *			
Life Purpose				
	Gender			
	Female	**1.40**	**1.16, 1.69**	**<0.001**
	Male *			
	Age			
	≤39 *			
	40–59	0.83	0.72, 0.95	0.007
	≥60	**0.55**	**0.47, 0.64**	**<0.001**
	Bond			
	Low *			
	Medium	**1.39**	**1.19, 1.62**	**<0.001**
	High	**2.95**	**1.50, 5.79**	**=0.002**
	Living arrangement			
	Live with others	**0.79**	**0.69, 0.90**	**=0.001**
	Live alone *			
Self-compassion				
	Gender			
	Female	**1.33**	**1.07, 1.65**	**=0.001**
	Male *			
	Age			
	≤39 *			
	40–59	**0.74**	**0.63, 0.87**	**<0.001**
	≥60	**0.55**	**0.46, 0.65**	**<0.001**
	Species			
	Cat	1.09	0.93, 1.28	=0.299
	Dog *			

* referent; OR, odds ratio; CI, confidence interval; bold denotes statistical significance.

## Data Availability

The raw data supporting the conclusions of this article will be made available by the authors, without undue reservation.

## Appendix A

**COVID-19 and cats**

Cat owners: COVID-19 is impacting all areas of our lives, including our relationships with our companion animals. We are researchers from Colorado State University, Washington State University, University of San Francisco, and Palo Alto University and we are interested in learning how COVID-19 has impacted your relationship with your cat. We are specifically looking for people who are over the age of 18 and are currently the primary caretaker of at least one cat. If you meet these qualifications, please consider taking the following short (~10 min) anonymous survey.
What is involved? You will be asked to complete a series of questions as honestly as possible and there are no right or wrong answers. The questionnaire should take no more than 10 min to complete. Participation is entirely voluntary. You may quit at any time.
Are there any benefits or risks in my taking part? There are no direct risks or benefits to completing the survey. The survey is voluntary and anonymous and you may stop the survey at any time by closing the window. Data from the survey will be used only for research and will hopefully be published in a journal.
Will my participation be confidential? Yes, all participation will be confidential. The data will be anonymous and will contain no information that could lead to the identity of individuals. Anonymous data will be kept on a password protected computer.
What happens if I change my mind? If you feel you do not wish to continue with the questionnaire, you can close the browser window.
Where can I get more information? If you have questions about this research please contact Dr. Lori Kogan (Lori.Kogan@ColoState.EDU). Any questions about participant rights related to this survey can be directed to CSU IRB (ricro_irb@mail.colostate.edu) or 970 491-1655. This study has been reviewed and approved by the Research Integrity and Compliance Review Office at Colorado State University.
Consent—I have read and understood the information given above. In consenting, I agree to take part in this research project and agree for my data to be used for the purpose of this study. I understand that my participation is voluntary and I may withdraw at any time.
Note: This survey asks questions related to COVID-19. If this topic causes any personal distress, you can stop the survey at any time by closing your browser. If you would like more information about COVID-19, please visit the CDC website: https://www.cdc.gov/.

Nearly every city is experiencing growing restrictions on travel, including those that have orders to stay at home except for food and essential services. As of today, what level of restrictions are currently in place in your city:

o No current restrictions
o Some stores/businesses and restaurants closed
o All non-essential stores/businesses closed but no orders to stay inside/stay at home
o All non-essential stores/businesses closed and order to stay inside/stay at home
o Other ________________________________________________

Please tell us a bit about your household in the following questions.

In what country do you currently live?

o United States
o Canada
o UK
o Australia
o Other (please specify) ________________________________________________

How many adults regularly live in your home (including yourself)?

▼ 1 ... >5

How many children (under 18 years of age) live in your home?

▼ 0 ... >5

How many cats live in your home?

▼ 1 ... >5

What is your age?

________________________________________________________________

Please indicate your gender:

o Male
o Female
o Non-binary
o NA

What is your current work status:

o I have always worked from home and am working from home now
o I was working outside the home and am now working from home
o I was working outside the home and am still working outside the home
o I was working outside the home and have been laid off
o I have always worked from home and have been laid off
o Other: ________________________________________________

Please think back to one month ago, right before the COVID 19 outbreak—how would you describe the availability of your social support system?

o Minimal available social support
o Some available social support
o A great deal of available social support

Please indicate the availability of your social support system at this time:

o Minimal available current social support
o Some available current social support
o A great deal of available current social support

Have you been diagnosed with COVID-19?

o Yes
o No

Do you suspect you have had (or currently have) COVID-19, but have not been tested?

o Yes (please explain why): ________________________________________________
o No

Have you been ordered to quarantine?

o Yes
o No

Have you placed yourself in self-quarantine?

o Yes
o No

For the following questions, if you have more than one cat, please answer the questions about the cat whose name starts with a letter closest to the beginning of the alphabet (for example, choose to answer about Fluffy rather than Sylvester since “F” comes before “S” in the alphabet).

Please think back to one month ago, right before the COVID 19 outbreak—how would you have rated your bond with your cat on a scale from 1 (not at all bonded) to 10 (extremely bonded)?

▼ 1 (not bonded at all) ... 10 (extremely bonded)

Now, thinking about today, how would you rate your bond with your cat on a scale from 1 (not at all bonded) to 10 (extremely bonded)?

▼ 1 (not bonded at all) ... 10 (extremely bonded)

Most cats have at least some behaviors that can be frustrating at times. Please think back to one month ago, right before the COVID 19 outbreak—how frustrated did you feel about your cat’s undesirable behavior(s) on a scale from 1 (minimal/no frustration) to 10 (extremely frustrated)?

▼ 1 (minimal/no frustration) ... NA (no undesirable behaviors)

Now, thinking about today, how frustrated do you feel about your cat’s undesirable behavior(s) on a scale from 1 (minimal/no frustration) to 10 (extremely frustrated)?

▼ 1 (minimal/no frustration) ... NA (no undesirable behaviors)

Please indicate the impact (if any) of COVID-19 on the time you spend with your cat (actively engaging or just being in the house together):

o Decreased amount of time
o Same amount of time
o Increased amount of time

Do you feel the increased amount of time you are spending with your cat is strengthening your relationship or creating strain in the relationship?

o Strengthening (please explain) ________________________________________________
o Straining (please explain) ________________________________________________
o Both strengthening and straining (please explain) ________________________________________________
o No change

Please indicate the impact of COVID-19 on the time you spend actively engaging with your cat in the house or yard (playing, petting, sitting/laying together):

o Decreased amount of time
o Same amount of time
o Increased amount of time

Please indicate how your cat has impacted EACH of the following feelings you might be having related to COVID-19.

	Greatly Decreased	Decreased	No Change/Impact	Increased	Greatly Increased	NA/Is Not an Issue
Feelings of anxiety	o	o	o	o	o	o
Feelings of depression	o	o	o	o	o	o
Feeling overwhelmed	o	o	o	o	o	o
Feeling isolated	o	o	o	o	o	o
Feeling lonely	o	o	o	o	o	o
Ability to maintain a regular schedule	o	o	o	o	o	o
Ability to cope with uncertainty	o	o	o	o	o	o
Give purpose/meaning to your life	o	o	o	o	o	o
Compassion towards myself	o	o	o	o	o	o

Please indicate your concern level with EACH of the following veterinary-related issues as they relate to COVID-19.

	No Concern	Minimal Concern	Some Concern	Great Concern	NA/Is Not an Issue
Ability to afford emergency veterinary care now	o	o	o	o	o
Ability to afford emergency veterinary care in the future	o	o	o	o	o
Ability to afford non-emergency veterinary care now	o	o	o	o	o
Ability to afford non-emergency veterinary care in the future	o	o	o	o	o
Concern that my vet will not be open/available if I need them for emergencies	o	o	o	o	o
Concern that my vet will not be open/available if I need them for non-emergencies	o	o	o	o	o
Concern about having to leave the house if my cat gets injured or sick	o	o	o	o	o

Please indicate your concern level with EACH of the following cat-related issues as they relate to COVID-19.

	No Concern	Minimal Concern	Some Concern	Great Concern	Na/Is Not an Issue
Ability to afford cat food/supplies now	o	o	o	o	o
Ability to afford cat food/supplies in the future	o	o	o	o	o
Ability to obtain (not due to financial reasons) cat food/supplies	o	o	o	o	o
A caretaker for your cat if you contracted COVID-19	o	o	o	o	o
Concern that you could give your cat COVID-19	o	o	o	o	o
Concern that your cat could give you COVID-19	o	o	o	o	o
Ability to play with your cat if you get COVID-19	o	o	o	o	o
Ability to keep your cat because of changes due to COVID-19	o	o	o	o	o

There are many stressors that can come with COVID-19. Do you feel that having a cat adds, reduces, or has no impact on your stress level? Please explain:

________________________________________________________________

Have you made a plan or identified someone who could care for your cat if you got sick?

o Yes
o No

Have you agreed to care for someone else’s cat if they got sick?

o Yes
o No, I have not been asked
o No, I have been asked, but I feel unable to commit to this
o Other: ________________________________________________

Has your cat needed veterinary care since the COVID 19 outbreak?

o Yes
o No

Have you taken your cat to the veterinarian since the COVID 19 outbreak?

o Yes
o No

How many times have you taken your cat to the veterinarian since the COVID 19 outbreak?

▼ 1 ... >5

Please indicate for what reason(s) you have taken your cat to the veterinarian since the COVID 19 outbreak? (select all that apply)

o Vaccinations
o Wellness exam
o Dental
o Skin/allergy
o Surgery
o Accident
o Vomiting/diarrhea
o Serious illness (e.g., cancer)
o Monitoring an illness/disease/recheck
o Euthanasia
o Other ________________________________________________

Were you allowed in the veterinary clinic for this appointment or did you interact in the parking lot/outside only?

o Allowed in clinic reception area and exam room
o Allowed in clinic reception area only
o Was met in the parking lot/outside, not allowed in veterinary clinic
o Other: ________________________________________________

Please describe your most recent veterinary visit experience since the COVID 19 outbreak:

________________________________________________________________

Please indicate your concern level, because of COVID-19 changes, about the ability to provide for **your own** basic needs (home, food, medical care, etc)?

o Not at all concerned
o Minimal concern
o Some concern
o A great deal of concern

Please indicate your concern level, because of COVID-19 changes, about the ability to provide for **your cat’s** basic needs (home, food, veterinary care, etc)?

o Not at all concerned
o Minimal concern
o Some concern
o A great deal of concern

Please share any additional thoughts about how COVID-19 as impacted your relationship with your cat:

________________________________________________________________

Have you adopted a cat since the COVID 19 outbreak?

o Yes
o No

Please explain why you decided to adopt a cat since the COVID 19 outbreak:

________________________________________________________________

When did you make the decision to adopt a cat?

o Before the COVID-19 outbreak
o Since the time of the COVID-19 outbreak

Do you plan to adopt a cat within the next month?

o Yes
o Maybe
o No

Please explain why you plan to adopt a cat in the next month:

________________________________________________________________

Have you fostered a cat since the COVID 19 outbreak?

o Yes
o No

Why have you chosen to foster a cat since the COVID 19 outbreak?

________________________________________________________________

Do you plan to foster a cat within the next month?

o Yes
o Maybe
o No

Why do you plan to foster a cat in the next month?

________________________________________________________________

When did you make the decision to foster a cat?

o Before the COVID-19 outbreak
o Since the time of the COVID-19 outbreak

Do you plan to continue fostering if you return to on-site work?

o I do not plan to return to on-site work
o Yes, I plan to continue fostering if I return to on-site work
o No, I do not plan to continue fostering if I return to on-site work
o Other (please explain) ________________________________________________

Before the COVID-19 outbreak, did you actively volunteer for an animal shelter/rescue (outside of fostering animals)?

o Yes
o No

After the COVID-19 outbreak, do you plan to volunteer for an animal shelter/rescue (outside of fostering animals)?

o Yes
o No

Now that you have completed the survey, please tell us anything else you would like to about how COVID-19 has impacted your relationship with your cat or your feelings about living with a cat.

________________________________________________________________

Thank you!
